# Reduced Necrotizing Enterocolitis after an Initiative to Promote Breastfeeding and Early Human Milk Administration

**DOI:** 10.1097/pq9.0000000000000014

**Published:** 2017-02-21

**Authors:** Michelle Feinberg, Lynn Miller, Barbara Engers, Kathy Bigelow, Ann Lewis, Shannon Brinker, Fran Kurland, Elizabeth Potthoff, Melynda Wallin, Alfonso Pantoja, John R. Britton

**Affiliations:** From the * Newborn Intensive Care Unit, Saint Joseph Hospital, Denver, Colo.; and † Department of Neonatology, Colorado Permanente Medical Group, Denver, Colo.

## Abstract

**Introduction:**

We sought to reduce the incidence of necrotizing enterocolitis (NEC) in premature infants (PI) by fostering the postnatal establishment of protective intestinal bacteria through early administration of human milk (HM) and probiotics.

**Methods:**

A multidisciplinary team implemented an initiative to support breastfeeding (BF) and provide early postnatal supplemental donor human milk (DHM) and probiotics to PI. Interventions included process improvements in milk preparation, storage, and fortification. PI admitted to our NICU between 2006 and 2015 were monitored for feeding of HM, DHM, and preterm formula (PF), frequency of early feedings, and incidence of NEC.

**Results:**

Retrospective review of 2557 cases revealed post-initiative increases in the percentage of PI receiving HM (91.5% to 96.1%), HM within 48 hours of birth (75% to 90.6%), and DHM (17.7% to 71.9%). The percentage of infants receiving feedings on day one increased from 23.9% to 44.6% while the percentage receiving PF within the first 72 hours declined (31.2% to 10.3%). The NEC rate declined from 4.1% to 0.4%. Reduction in NEC occurred despite a simultaneous increase in perinatal antibiotic exposure and the universal but late administration of bovine HM fortifier. The improvement associated with the decrease in NEC included initiation of probiotic administration, a reduction in PF feeding, and improvements in milk preparation, storage, and fortification processes.

**Conclusions:**

Early exclusive feedings of HM and avoidance of PF together with probiotics and milkhygiene may decrease NEC in PI. Neither brief perinatal antibiotic exposure nor late introduction of bovine fortifiers appears detrimental in this context.

## INTRODUCTION

For premature infants (PI), human milk (HM) feeding reduces the risk of retinopathy of prematurity, sepsis, and necrotizing enterocolitis (NEC).^1–3^ However, mothers who deliver PI are often unable to initiate and maintain lactation. Poor lactation results in insufficient milk for their infants, especially during the first postnatal week.^4,5^ Because early enteral nutrition is important to promote gastrointestinal growth and development,^6^ delayed introduction of enteral feedings may increase the time to establish full enteral feedings and may prolong the need for parenteral nutrition with its associated morbidities.^7^

Bovine milk preterm formula (PF) is often used to supplement limited maternal milk supplies.^8^ However, some investigators associate PF feeds with an increased risk of NEC^9–11^ and prefer donor human milk (DHM) feedings instead.^12,13^ PI fed DHM have reduced rates of NEC compared with those fed PF.^14,15^ Moreover, Kantorowska et al^12^ associate the increased availability of DHM with decreasing rates of NEC.

A potential mechanism for the pathogenesis of NEC is the failure to establish a population of normal intestinal commensal bacteria in the presence of an immature gut mucosal barrier and underdeveloped systemic immune system. This combination may lead to a proinflammatory state that allows intestinal damage with translocation of pathogens across the mucosa.^16,17^ Conversely, the early postnatal establishment of a beneficial population of commensal bacteria may protect the premature intestine from inflammation and injury.^16^ HM fosters the development of commensal bacteria and may prevent NEC by this mechanism.^10,18^ Probiotic administration may reduce NEC by a similar mechanism.^17^

In light of the benefits of early enteral feedings and the advantages of HM over PF for PI, we embarked upon a quality improvement (QI) initiative to increase breastfeeding (BF), facilitate early HM feedings, and provide DHM to supplement limited maternal milk supplies for PI in our newborn intensive care unit (NICU). Additional interventions included the early introduction of probiotics and hygienic changes in milk preparation, storage, and fortification processes. We focused on the first several days after birth, hypothesizing that early introduction of HM feedings and probiotics, in preference to deferral of feedings or early PF, would reduce the rate of NEC by fostering development of a beneficial population of commensal bacteria.

## METHODS

### Context

We conducted this QI initiative in the level IIIB NICU at St. Joseph Hospital in Denver, Colorado, after approval by the institutional review board. The NICU has a decade history of multiple ongoing multidisciplinary QI initiatives. The project included all inborn PI admitted to the NICU between January 1, 2006, and December 31, 2015.

### Process Changes and Interventions

The initiative was conducted by a committee consisting of registered nurses, a dietitian, an occupational therapist, a lactation consultant, nutrition technicians, a neonatal nurse practitioner (NNP), and a neonatologist. Our general approach is extensively described in previous publications.^19,20^

Since our goal was a reduction in NEC, the committee first conducted literature reviews of risk factors for NEC and potential measures for its prevention. The reviews took the form of journal clubs to which all neonatologists and NNPs were invited. They suggested that early HM and probiotics might prevent NEC. We also deemed avoidance of early PF and H2 blockers and general hygiene in the milk preparation, storage, and fortification processes potentially relevant by limiting exposure to bovine milk proteins and potential pathogens.

Analyses of feeding practices indicated that BF rates were already high, but first feedings often began several days after birth and early maternal milk feedings were frequently supplemented with PF. All BF PI received bovine milk product fortification at ≥24 calories/ounce after the fifth day. H2 blocker use was very low, discouraged in our unit by consensus in 2009. However, milk preparation, storage, and fortification were performed by bedside nurses in a nonuniform manner with variable precautions for maintaining sterility.

Accordingly, we formulated 4 objectives for process change: (1) to increase HM feeding by encouragement and support of BF; (2) to provide DHM to supplement maternal milk in preference to PF or deferred feedings from birth until attainment of adequate maternal lactogenesis; (3) to supplement early HM feedings with probiotics; and (4) to standardize processes for milk preparation, storage, and fortification.

We formulated process changes into procedural guidelines that were reviewed, revised, and incorporated into formal policies. Interventions to accomplish these process changes were targeted toward 3 groups: (1) neonatologists and NNPs, who were responsible for ordering feedings and probiotics; (2) parents, who chose whether or not to breastfeed and to consent to use of DHM for their infants; and (3) staff, who implemented process changes.

Interventions for neonatologists and NNPs included journal club participation, participation in policy formulation, and group review of options for dietary and probiotic orders in the electronic medical record. We describe parental interventions for the first 2 objectives below. Interventions for staff fostered implementation of process changes according to corresponding guidelines using a standardized approach as follows. During an educational phase, we presented each guideline via posters, e-mail messages, and verbal presentations. Surveys assessed understanding of the process changes and means for implementation. Implementation proceeded according to its guideline on a predetermined date and made frequent use of rapid small-scale cycle improvement (plan-do-study-act). Audits of eligible patients were performed every 2–3 weeks, and the percentage of eligible patients for which the change was successfully made was calculated; audits continued until compliance exceeded 90%. Feedback that included audit results was presented to all staff during the study phase of plan-do-study-act cycles, usually in organized group sessions, with ongoing education, encouragement, and support.

We pursued each objective as follows:

*First objective*: Mechanisms to encourage and support BF began in the years 2010 and 2011 and included universal lactation consultation, development of a customized breast-pumping log, and a brochure and video presentation on BF for parents.

*Second objective*: Beginning in 2006, we developed a consent form for DHM administration. Pasteurized DHM was obtained from the Mothers’ Milk Bank, Arvada, Colorado, and initiated as a supplement to maternal milk during the first 1–2 days after birth. From 2006 to 2010, select very low birth weight (VLBW) infants received DHM on a limited basis; in February 2011, the amount was increased to 12 ounces for all PI and in July 2012 to unlimited free amounts. The duration and amount of DHM received daily varied. Appropriate standardized feeding orders in the electronic medical record facilitated these changes.

*Third objective*: Probiotics (Risaquad-2 Double Strength, 1–2 billion colony-forming units daily from the day of birth until hospital discharge, Rising Pharmaceuticals, Inc., Allendale, N.J.) for infants less than 34 weeks of gestation or less than or equal to 1,500 g began in July 2013. Probiotic use was extended to all PI with an anticipated NICU stay of greater than 72 hours starting in July 2015. Incorporation into admission orders in the electronic medical record facilitated these changes.

*Fourth objective*: The groundwork for some of these changes began as early as 2012, but the full implementation did not occur until November to December 2014. These included (1) adoption of a standardized process for milk preparation at a designated location by a gowned and gloved provider; (2) standardized warming of milk before feeding using a milk warmer; (3) elimination of all powdered supplements with a transition to a liquid bovine preterm fortifier and liquid protein supplement (Similac Human Milk Fortifier Concentrated Liquid and Liquid Protein Fortifier, respectively, Abbott Nutrition, Columbus, Ohio). Since December 2014, fortified milk has been prepared only by certified nutrition technicians in a fully dedicated preparation room in a new hospital under aseptic conditions.

### Analysis

We extracted data from a computerized medical record. Baseline (2006–2009) and postintervention (2013–2015) periods were defined, and process changes (receipt of HM, HM within 48 hours, DHM, PF, and feedings on the day of birth) were compared for both periods. A run chart was used to monitor outcome (NEC) changes, with the percentage of infants with NEC on the ordinate and the year of birth on the abscissa. NEC was defined according to the Bell criteria,^21^ and only patients with stage II or III disease were deemed to have the condition.

Bivariate analyses included the chi-square test for categorical variables, the *t* test for continuous variables, and Pearson correlations. Binary logistic regression assessed factors associated with NEC. Statistical analyses utilized SPSS (SPSS, Chicago, Ill.), with significance accepted at *P* < 0.05.

## RESULTS

During the entire period studied, 2,557 PI were admitted to the NICU (annual mean number ± SD: preterm = 255.6 ± 28.2, VLBW = 62.1 ± 13.7). Receipt of HM, HM within 48 hours, DHM, and feedings on the day of birth increased, whereas receipt of PF within the first 72 hours decreased in the postintervention period compared with the baseline period (objectives 1 and 2, Table 1).

**Table 1.**
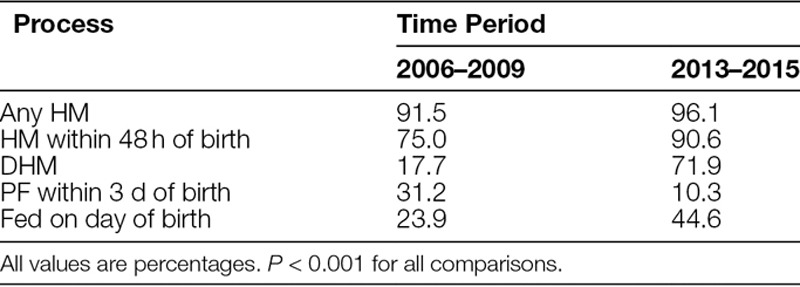
Process Changes by Time Period

**Fig. 1.**
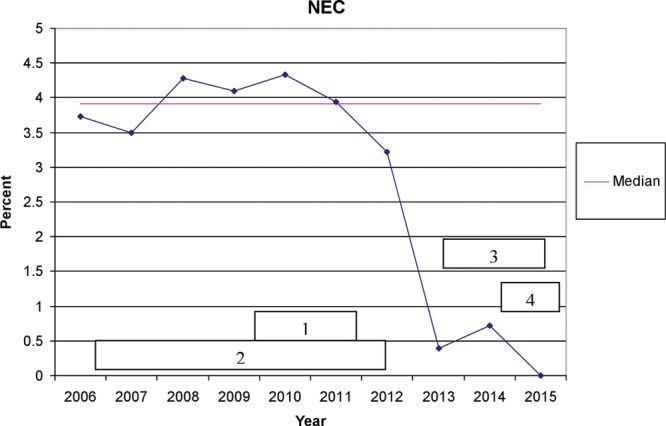
Run chart of the percentage of PI admitted to the NICU who developed NEC. Numbered horizontal bars indicate timing of interventions associated with corresponding objectives described in the Methods section.

The incidence of NEC declined during 2013 to 2015 compared with that during 2006 to 2009 (0.4% vs 4.1%; *P* < 0.001; Fig. 1). NEC rates declined from 8.3% to 1.0% for VLBW infants and from 2.4% to 0.2% for non-VLBW infants (*P* < 0.001 for both groups). During the entire period, half of the NEC cases occurred among VLBW infants and half in PI > 1,500 g.

To explore other potential contributions to the reduction in NEC, we compared PI born during the 2 periods on a wide variety of characteristics (Table 2). Compared with baseline infants, postintervention infants were more likely to be female, receive prenatal steroids and perinatal antibiotics, be born after pregnancies complicated by maternal hypertension, and have lower 5-minute Apgar scores. They were less likely to receive H2 blockers, reflecting our conscious effort to eliminate the use of these agents in 2009. Review of individual cases of NEC revealed none receiving them before the onset of the disease, but some received the drugs for post-NEC problems.

**Table 2.**
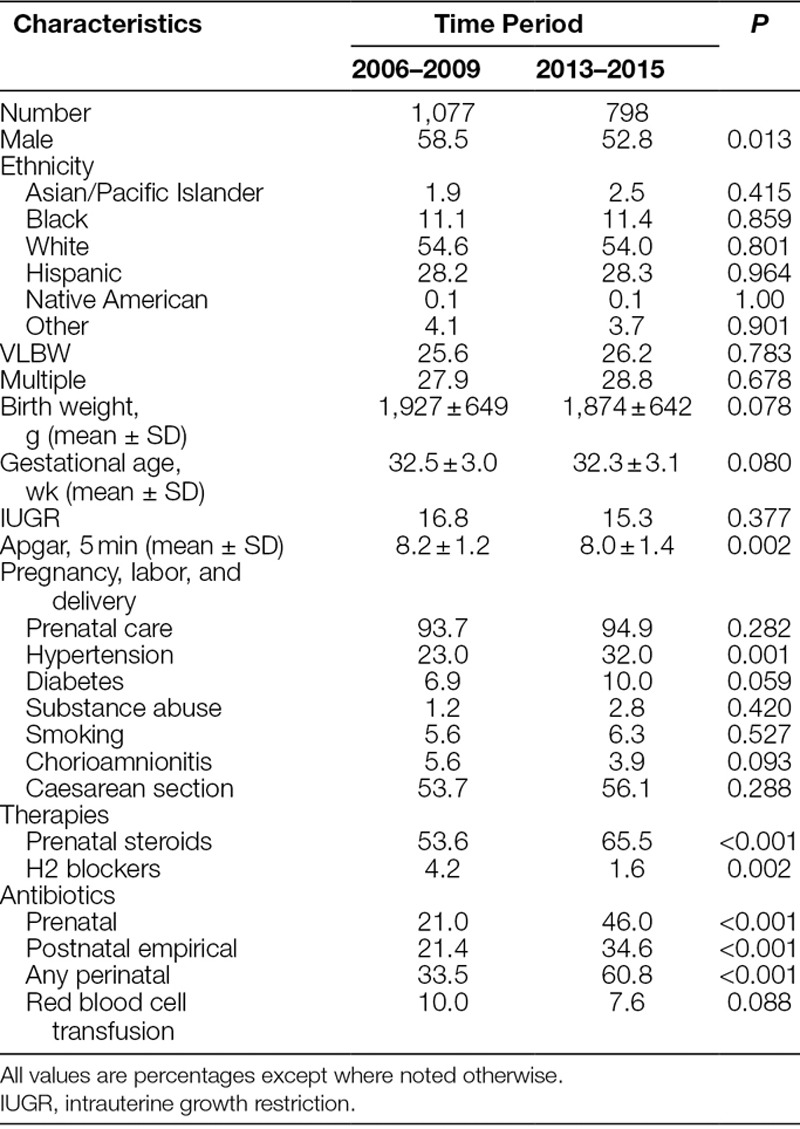
Population Characteristics by Time Period

To evaluate the potential factors associated with NEC during the different time periods, we analyzed the characteristics of the entire population with and without NEC. Lower gestational age, lower birth weight, and receipt of prenatal steroids were all significantly associated with NEC (Table 3). As expected, these variables correlated inversely with gestational age: prenatal steroids (r = −0.54), birth weight (r = −0.80), both with *P* ≤ 0.001. Entering gestational age, the major known predictor of NEC, as an independent variable into a logistic regression model with NEC as the outcome yielded an unadjusted odds ratio of 0.79, with 95% confidence interval 0.74 to 0.85, *P* < 0.001. None of the other variables in Table 3 contributed significantly to this model when entered singly or in combination. Therefore, we conclude that none of the factors that differed between the 2 time periods was independently associated with the decline in NEC.

**Table 3.**
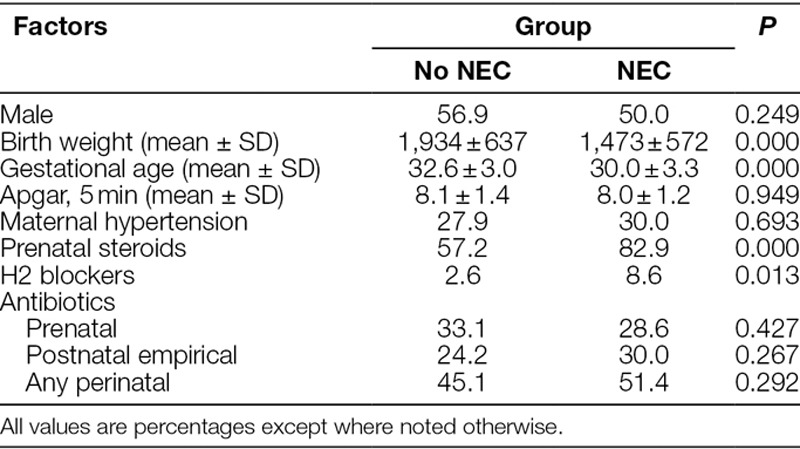
Characteristics of Patients With and Without NEC (2006–2015)

## DISCUSSION

We reduced NEC in our population of PI after successful efforts to promote BF and early use of HM. Probiotics and changes in milk preparation, storage, and fortification processes may have sustained the low incidence.^22–25^

In our preterm population, perinatal antibiotic exposure increased at a time when NEC declined. Prolonged duration of initial empirical antibiotic therapy is thought to decrease bacterial diversity, reduce colonization of beneficial bacteria, promote the growth of potential bacterial pathogens,^17^ and lead to NEC in PI.^26^ However, prolonged empirical antibiotic administration for greater than or equal to 5 days, as previously described in association with NEC,^26,27^ was extremely uncommon in our unit during the entire time period. In contrast, it is standard practice in our unit to discontinue initial empirical antibiotics after blood cultures remain negative for 24 to 48 hours as determined by a rapid detection monitoring system.^28^ Similarly, intrapartum antibiotic exposure, which also increased during the period of declining NEC in our population, is also typically brief. Moreover, previous reports of NEC associated with early antibiotics were from populations with unreported^26^ or low^27^ rates of HM feeding compared with ours. Possibly, the relatively brief perinatal antibiotic exposure in our population was either not detrimental in the setting of early HM feedings or even beneficial in reducing NEC. In this regard, oral antibiotics have been found to reduce the incidence of NEC in PI,^29^ and early brief antibiotic treatment has been associated with reduced rates of NEC in animal models.^30^

Previous workers^10,11,31^ reported lower rates of NEC in infants fed HM-based diets exclusively compared with those fed PF or a combined diet of HM and bovine milk–based products. In addition to receiving less HM, infants receiving bovine milk products are exposed to bovine proteins, some of which produce NEC in animal models.^32^ In our population, a dramatic decline in NEC occurred coincident with declining use of early PF (<3 days), yet continued universal use of bovine milk–based fortifiers (>5 days). Possibly, avoidance of early PF, combined with the protective effects of early HM and probiotics, fostered a state invulnerable to potential insult by subsequently encountered bovine fortifier proteins. In light of the continued high use of bovine milk–based fortifiers in our unit and the absence of NEC, it is hard to implicate these agents as pathophysiologic in the present context. Consistent with this conclusion is the report of Schanler et al, which suggested that maternal milk with bovine milk–based fortifier was protective against the combined incidence of sepsis and NEC.^33^

Our magnitude of NEC reduction compares favorably with previous reports of reduced incidence of NEC with HM feedings compared with PF feedings. Hair et al^9^ observed a decline from 16.7% to 6.9%, Abrams et al^11^ a decline from 17% to 5%, and Cristofalo et al^15^ a decline from 21% to 3%. For the combined outcome of NEC or death, Sullivan et al^31^ observed a decline from 20% to 6%. All of these studies included only infants less than 1,250 g at birth. In contrast, our rate of NEC declined from 4% to 0.4% for all PI and from 8.3% to 1.0% for VLBW infants.

Limitations of the present study include its retrospective analysis and implementation of process changes at different time periods in a single NICU. Timing and magnitude of the decline in NEC did not precisely align with that of most of the process changes. Increases in overall BF, early feedings, and early administration of HM were modest and loosely associated with the decline in NEC. Also, other unmeasured factors may have contributed to the decline.

Nevertheless, the observed decline in NEC in our unit was dramatic. We speculate that (1) early introduction of HM with or without probiotics fostered establishment of a beneficial population of commensal bacteria that protected the premature intestine from inflammation and injury, leading to reduction in the risk of NEC; (2) coincident receipt of brief perinatal antibiotic therapy was either not detrimental or may have contributed additional benefit; and (3) subsequent receipt of bovine milk fortifiers was not detrimental because it occurred after establishment of a population of beneficial flora. These hypotheses deserve further exploration in future randomized trials.

## DISCLOSURE

The authors have no financial interest to declare in relation to the content of this article. This study was supported by departmental resources.
